# A spectrum of free software tools for processing the VCF variant call format: vcflib, bio-vcf, cyvcf2, hts-nim and slivar

**DOI:** 10.1371/journal.pcbi.1009123

**Published:** 2022-05-31

**Authors:** Erik Garrison, Zev N. Kronenberg, Eric T. Dawson, Brent S. Pedersen, Pjotr Prins

**Affiliations:** 1 Department Genetics, Genomics and Informatics, University of Tennessee Health Science Center, Memphis, Tennessee, United States of America; 2 Pacific Biosciences, San Diego, California, United States of America; 3 NVIDIA Corporation, Santa Clara, California, United States of America; 4 Center for Molecular Medicine, University Medical Center, Utrecht, The Netherlands; Hebrew University of Jerusalem, ISRAEL

## Abstract

Since its introduction in 2011 the variant call format (VCF) has been widely adopted for processing DNA and RNA variants in practically all population studies—as well as in somatic and germline mutation studies. The VCF format can represent single nucleotide variants, multi-nucleotide variants, insertions and deletions, and simple structural variants called and anchored against a reference genome.

Here we present a spectrum of over 125 useful, complimentary free and open source software tools and libraries, we wrote and made available through the multiple vcflib, bio-vcf, cyvcf2, hts-nim and slivar projects. These tools are applied for comparison, filtering, normalisation, smoothing and annotation of VCF, as well as output of statistics, visualisation, and transformations of files variants. These tools run everyday in critical biomedical pipelines and countless shell scripts. Our tools are part of the wider bioinformatics ecosystem and we highlight best practices.

We shortly discuss the design of VCF, lessons learnt, and how we can address more complex variation through pangenome graph formats, variation that can not easily be represented by the VCF format.

## Author summary

Most bioinformatics workflows deal with DNA/RNA variations that are typically represented in the variant call format (VCF)—a file format that describes mutations (SNP and MNP), insertions and deletions (INDEL) against a reference genome. Here we present a wide range of free and open source software tools that are used in biomedical sequencing workflows around the world today.

This is a *PLOS Computational Biology* Software paper.

## 1 Introduction

From its introduction in 2011 the VCF variant call format has become pervasive in bioinformatics sequencing workflows [1, 2]. VCF is one of the important file formats in bioinformatics workflows because of its critical role in describing DNA and RNA variants. VCF can describe single- and multi- nucleotide polymorphisms (SNP & MNP), insertions and deletions (INDEL), and simple structural variants (SV) against a reference genome [1]. Practically all important variant callers, such as GATK [3] and freebayes [4], produce files in the VCF format. The VCF file format is used in population studies as well as somatic mutation and germline mutation studies. In this paper we discuss the tools we wrote to process VCF and we shortly discuss strengths and shortcomings of the VCF format. We discuss how we can improve future variant calling in its contribution to population genetics.

An important part of the success of VCF that it is a relatively simple and flexible standard that is easy to read, understand and parse. This feature has resulted in wide adoption by bioinformatics software developers. VCF typically scales well in bioinformatics workflows because files can be indexed [5], compressed [1, 6, 7] and trivially parallelized in workflows by splitting files and processing them independently, e.g. [4].

Here we present and discuss a spectrum of important and complementary tools and libraries we wrote for processing VCF in sequencing workflows, i.e., vcflib, bio-vcf, cyvcf2, slivar and hts-nim. These tools were created by the authors for the demands of large VCF file processing and data analysis following the Unix philosophy, as explained in the ‘small tools manifesto’ [8]. Development of these tools was often driven by the need to transform VCF into other formats, to digest information, to address quality control, and to compute statistics. The vcflib toolkit contains both a library and collection of executable programs for transforming VCF files written in the C++ programming language for performance. bio-vcf and slivar are domain-specific languages (DSL) for convenient querying and transforming VCF. cyvcf2 is a python library [9]. hts-nim is written in the compiled Nim language [10]. These are all useful tools and libraries for VCF processing that can pipe into each other for advanced processing.

## 2 Design and implementation

### 2.1 vcflib C++ tools and libraries

The vcflib toolkit contains both a library and collection of executable programs for transforming VCF files written in the C++ programming language for performance. vcflib includes a toolkit for population genetics: the Genotype Phenotype Association Toolkit (GPAT). These ransformations and statistical functions provided in the toolkit were written for the requirements of projects such as the The 1,000 Genomes Project [11] and NIST’s genome in a bottle [12].

**Fig 1:**
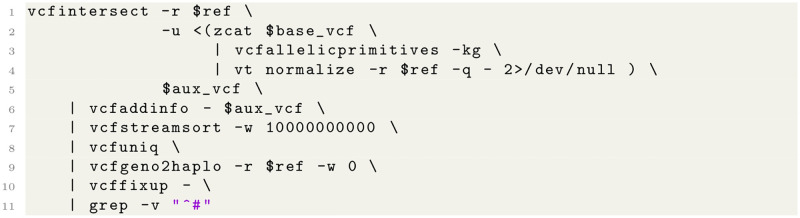


At its core, vcflib provides C++ tools and a library application programmers interface (API) to plain text and compressed VCF files. A collection of 83 command line utilities is provided, as well as 44 command line scripts (see Table 1). Most of these tools are designed to be strung together: piping the output of one program into the next, thereby preventing the creation of intermediate files, parallelize processing, and reducing the number IO operations. For example, the vcfjoincalls shell script includes the pipeline shown in Fig 1 (see Table 1 for a short description of the individual vcflib steps; vt is a variant normalization tool [13]):

**Table 1:** A selection of VCF processing tools included with vcflib (a full list of over 125 tools with full descriptions and options can be found in the online vcflib documentation [17]).

Name	Description
**Tools for filtering**	
vcfaddinfo	add info fields from a second VCF file for records missing in the first file.
vcffixup	insert AC and NS fields using sample genotypes
vcffilter	filter on common fields, e.g. *DP* > 10
vcfuniq	filter out duplicate entries
vcfuniqalleles	filter unique alleles only
**Tools for transformation**	
vcfintersect	set operations—intersect, union, complement
vcfstreamsort	sort
vcfoverlay	merge files by overlaying
vcfcombine	combine samples on identical sites
vcffixup	update fields
vcfannotate	annotate records from BED file
vcfflatten	flatten multi-allelic sites with common ALT genotype
vcfgeno2haplo	transform phased alleles into haplotypes
vcfsamplediff	compare VCF files and add annotations to INFO
vcfprimers	design primers
vcf2tsv	convert to tab separated table
vcf2fasta	convert to FASTA
vcf2bed	convert to BED
vcf2sqlite	convert to SQLite
smoother	averages a set of scores over a sliding genomic window
**Tools for metrics**	
vcfdistance	compute distance between positions and add field
vcfentropy	annotates and add field for sequence entropy for a window
**Tools for genotyping**	
vcfallelicprimitives	split records if multiple allelic primitives (gaps or mismatches) are specified in a single VCF record
genotypeSummmary	summarizes genotype counts for bi-allelic SNVs and INDEL
hapLRT	likelihood ratio test for haplotype lengths
**Tools for statistics**	
iHS	integrated ratio of haplotype decay between reference and non-reference allele
vFst	compute vFst as a measure of CNV stratification
vcfroc	compute a pseudo-ROC curve
meltEHH	plot extended haplotype homozygosity (EHH) curves
plotHaps	provide output for haplotype plots
popStat	population genetic statistics for each SNP
vcfrandomsample	random sampling
**Tools for validation**	
vcfcheck	check integrity and identity against reference genome

#### 2.2.1 The evolving VCF textual format

**Fig 2:**
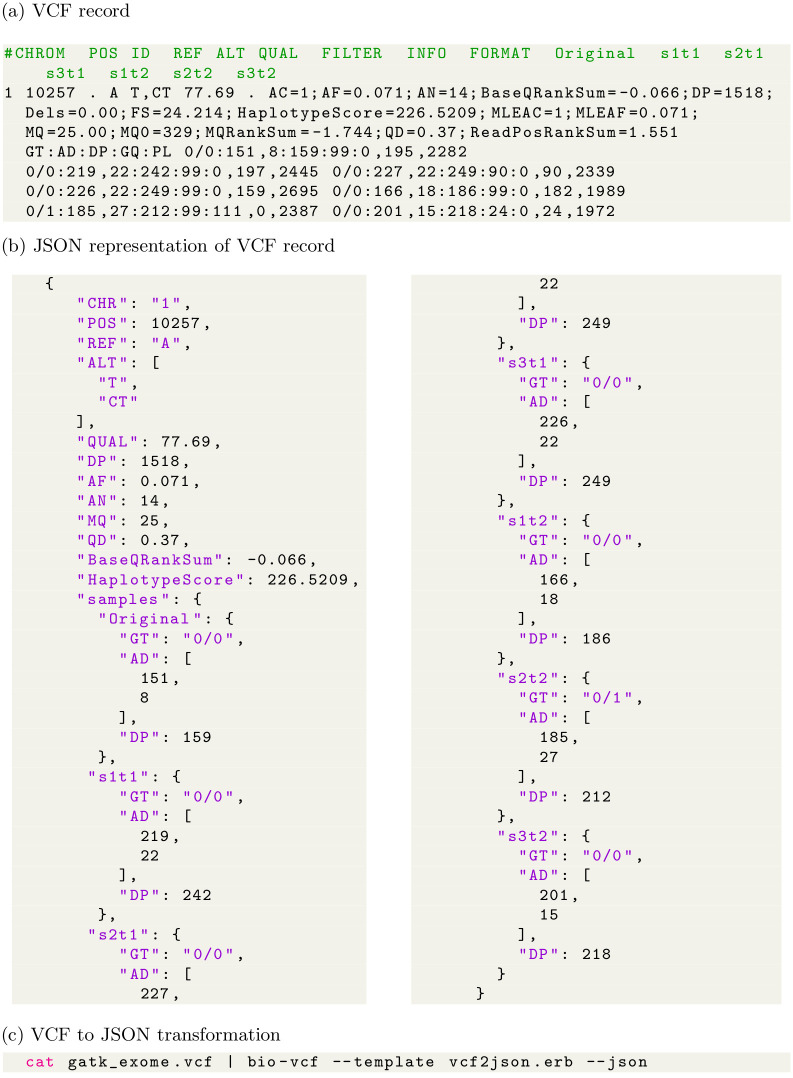
Example of the VCF format and a VCF transformation to Javascript Object Notation (JSON) using bio-vcf. (a) the line-based VCF record makes use of separators to split tab-delimited fields into subfields. Subfields are split with characters, =:;/ and so on. This splitting effectively projects a ‘tree-like’ datastructure that can also be represented as (b) a JSON record. JSON is used as a common data exchange format for databases and web-services. This example was generated with (c) the bio-vcf tool using a template [24]. bio-vcf transform data to any textual format, including RDF, HTML, XML etc. See also the bio-vcf section.

The VCF format is a textual file format: each line line describes a variant, i.e., a single nucleotide variant (SNV), an insertion, a deletion or a structural variant with rich annotation [1]. In a VCF line, fields are separated by the TAB character. Fields for chromosome, position, the reference sequence, the ALT alleles, and fields for quality, filter, INFO, FORMAT and calls for multiple samples are expected (see Fig 2). To split fields, for example for ‘ALT T,CT’ another separator is used; in this case a comma. VCF makes use of many separators by splitting fields into subfields, subsubfields and so on: effectively projecting a ‘tree’ datastructure onto a single line. The advantage is that it is easy to view a VCF file and it is almost trivial to write a basic VCF parser and it is easy to add information to VCF, sometimes leading to unwieldy nested annotations. The tree-structure is also the reason VCF files do not easily fit into spreadsheets. An evolving VCF ‘standard’ is tracked by the samtools/htslib project [14] and later amendments are particularly focused on more complex structural arrangements of DNA/RNA with ALT fields taking somewhat *ad hoc* creative forms, such as ‘A[3:67656[’ combined with an INFO field containing ‘SVTYPE = BND’ meaning that starting at reference position on a different chromosome, an ALT A nucleotide is followed by the sequence starting at chromosome 3 and position 67656. These SV annotations do away with some of the original simplicity of VCF. There are many such exensions introduced since the first publication of the VCF ‘standard’ that are used by specific SV and liftover/multiref tools (e.g. DVCF [15] and spVCF [16]) and largely ignored by most VCF processing tools, though our generic DSLs bio-vcf and slivar should be able to parse them.

## 3 Results

### 3.1 vcflib application programming interface (API)

The vcflib API describes a class vcflib::VariantCallFile to manage the reading of VCF files, and vcflib::Variant to describe the information contained in a single VCF record. The API provides iterators that are used in every included tool. For every record the tree-type hierarchy (Fig 2) of information can be navigated in the record through interfaces to the fixed fields (CHROM, POS, ID, QUAL, FILTER, INFO) and sample-related fields (FORMAT, and samples). vcflib implements functions for accessing and modifying data in these fields; interpreting the alleles and genotypes in record; filtering sites, alleles, and genotypes via a domain-specific filtering boolean language; and reading and writing VCF streams which are shared and used by all included tools.

### 3.2 vcflib command line tools

Table 1 short lists some important vcflib tools. A full list of tools is documented on the vcflib website [17]. Just to show the diversity of tools that can be explored we give a few examples: vcflib includes a wide range of tools for transformations, e.g., vcf2dag converts VCF to a partially ordered directed acyclic graph (DAG). vcfannotate intersects the records in the VCF file with targets provided in a BED file and vcfannotategenotypes annotates genotypes in the first file with genotypes in the second. vcfclassify generates a new VCF where each variant is tagged by allele class: SNP, Ts/Tv, INDEL, and MNP. vcfglxgt sets genotypes using the maximum genotype likelihood for each sample. vcfinfosummarize and vcfsample2info edit annotations given in the per-sample fields and adds the mean, median, min, or max to the site-level INFO. vcfleftalign Left-align INDELs and complex variants in the input using a pairwise REF/ALT alignment followed by a heuristic, iterative left realignment process that shifts INDEL representations to their absolute leftmost (5’) extent. vcflib includes tools for genotype detection. For example, the aptly named abba-baba tool calculates the tree pattern for four individuals with one ancestral reference.

### 3.3 vcflib tools for genome-wide association (GWA)

vcflib includes statistical GWA tools for phenotype association straight from VCF files. We have developed a flexible genotype-phenotype software library designed specifically for large and noisy NGS datasets. A mixture of traditional and novel population genetic methods have been implemented in the Genotype Phenotype Association Toolkit (GPAT++) part of vcflib. For example, permuteGPAT++, adds empirical p-values to a GPAT++ score. And vcfld computes linkage disequilibrium (LD). GPAT++ includes basic population stats (Af, Pi, eHH, oHet, genotypeCounts) and several flavors of Fst and tools for linkage, association testing (genotypic and pooled data), haplotype methods (hapLrt), smoothing, permutation, and plotting.

**Fig 3:**
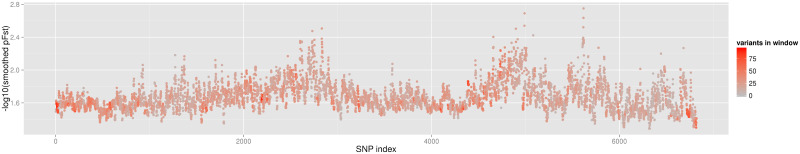
Smoothed pFst (−*log*10) statistic with color coded number of variants in a window. As computed by vcflib’s pFst and smoother tools [17].

For example, Wright’s F-statistics provide important insights into the evolutionary processes that influence the structure of genetic variation within and among populations (Fig 3) [18]. Fst is defined as the correlation between randomly sampled gametes relative to the total drawn from the same subpopulation [19]. wcFst is Weir & Cockerham’s Fst for two populations [20]. pFst is a probabilistic approach for detecting differences in allele frequencies between two populations and bFst is a Bayesian approach. bFst accounts for genotype uncertainty in the model using genotype likelihoods [18]. With vcflib the likelihood function has been modified to use genotype likelihoods provided by variant callers. There are five free parameters estimated in the model: each subpopulation’s allele frequency and Fis (fixation index, within each subpopulation), a free parameter for the total population’s allele frequency, and Fst. pVst calculates *Vst* to test the difference in copy numbers at each SV between two groups: *Vst* = (*Vt* − *Vs*)/*Vt*, where *Vt* is the overall variance of copy number and *Vs* the average variance within populations. sequenceDiversity calculates two popular metrics of haplotype diversity: *Pi* and extended haplotype homozygosity (eHH). *Pi* is calculated using the Nei and Li formulation [21]. eHH is a convenient way to think about haplotype diversity. When eHH = 0 all haplotypes in the window are unique and when eHH = 1 all haplotypes in the window are identical. The vcfremap tool attempts to realign, for each alternate allele, against the reference genome with a lowered gap open penalty and adjusts the CIGAR and REF/ALT alleles accordingly.

vcflib includes tools for genotype statisics. vcfgenosummarize adds summary statistics to each record summarizing qualities reported in called genotypes. It uses RO (reference observation count), QR (quality sum reference observations) AO (alternate observation count), QA (quality sum alternate observations). The normalizeHS is used for iHS and XP-EHH scores [22].

A full list of over 125 vcflib commands and functionality can be found on the website, as well as documentation and examples of application [17].

### 3.4 Bio-vcf and Slivar flexible command-line DSL filters and transformers

#### 3.4.1 bio-vcf

**Fig 4:**
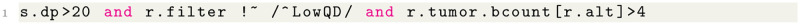


Compared to vcflib with its many individual dedicated command line tools, bio-vcf takes a different approach by providing a single command line tool that uses a domain specific language (DSL) for processing the VCF format. Thanks to a dynamic interpretation of the VCF tree representation (see Fig 2) all data elements in a VCF header or record can be reached using field names and their sub names. For example, Fig 4 is a valid select filter:

which selects all variants where the sample depth field s.db is larger than 20, where the FILTER field of a record r does not start with the letters *LowQD* (note it uses a Perl/Ruby-style regular expression or regex [23]), and where the tumor bcount of the *ALT* allele is larger than 4. The letter ‘r’ represents a record or line in a VCF file and the letter ‘s’ stands for each sample in a record.

**Fig 5:**
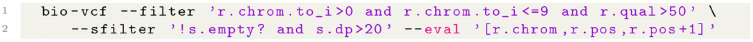


**Fig 6:**
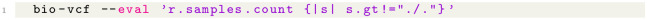


The naming of variables, such as s.dp and r.tumor.bcount, is inferred from the VCF file itself, so if a VCF has different naming conventions they are picked up automatically.

bio-vcf typically reads from the terminal STDIN and writes to STDOUT. The following full command line invocation reads VCF files and filters for chromosomes 1–9 where the quality (r.qual) is larger than 50. It also checks for non-empty samples where the sample read depth is larger than 20. For each selected record with --eval it outputs a BED record (the default output is the VCF record itself, useful for filtering) (Fig 5):

For comparisons and for output, fields can be converted to integers, floats and strings with to_i, to_f and to_s respectively. Note that these are Ruby functions and, in fact, all such Ruby functionality is available in bio-vcf statements. For extreme flexibility bio-vcf even supports *lambdas* which makes for very powerful queries and transformations. For example, to output the count of valid genotype fields in samples one could use the command shown in Fig 6,

where count is a function that invokes the *lambda* s.gt!=“./.”, i.e., where the genotype gt of sample s is not equal to “./.”. Sample ‘s’ is passed as a parameter.

Because of the flexibility of bio-vcf almost all imaginable data queries can be executed. bio-vcf was implemented for processing large VCF files and is fast because it is designed make use of multi-core processors (using Linux parallelized copy-on-write, i.e. a technique where RAM is shared between processes). bio-vcf is also ‘lazy’ which means that it only parses fields when they are used. For example, in the above query, only the sample *GT* field is unpacked and parsed to get a result. All other data in the record is ignored by the query and not evaluated. This contrasts largely with most VCF parsers in use today.

**Fig 7:**
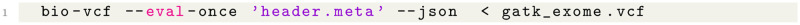


**Fig 8:**
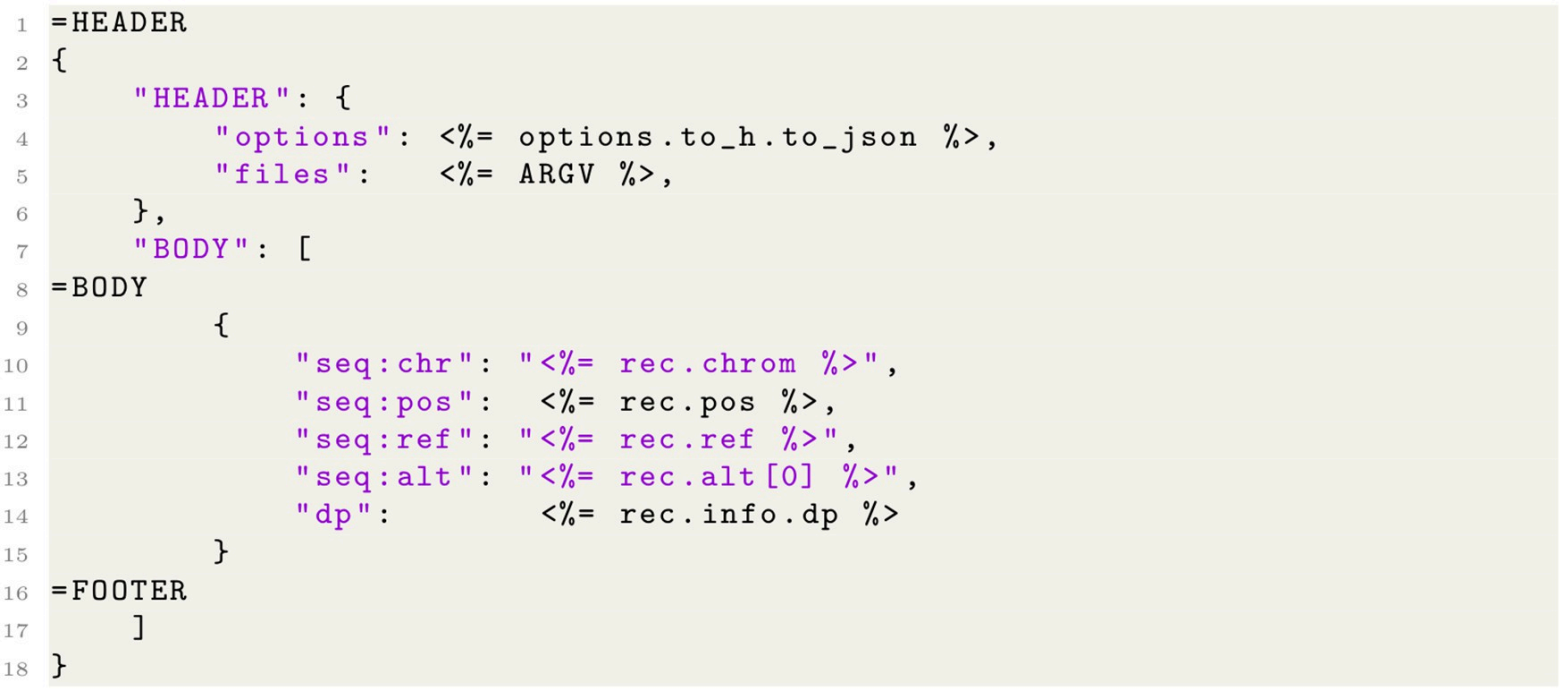


**Fig 9:**
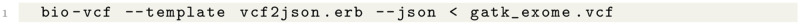


Finally, bio-vcf comes with a full parser and lexer that can tokenize the VCF file header and transform that in some other format. For example, the command in Fig 7

will turn the metadata information passed by GATK [3] into a JSON document. To get a full JSON document of the VCF file use a template that looks like Fig 8,

and run the command shown in Fig 9.

The high expressiveness and adaptable parsing makes bio-vcf a very powerful tool for searching, filtering and rewriting VCF files. See the bio-vcf website for full information on record and sample inclusion/exclusion filters, generators, multicore performance, field computations, statistics, genotype processing, set analysis and templates for user definable output, including templates for output of VCF header information and records for RDF, JSON, LaTeX, HTML and BED formats [24].

#### 3.4.2 Slivar

**Fig 10:**
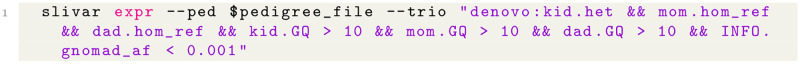


Similar to bio-vcf, slivar allows users to specify simple expressions for filtering and annotation [25]. Whereas bio-vcf uses Ruby to supply the DSL, slivar uses Javascript. slivar has built-in pedigree support for the samples so that, for example, a single expression can be applied to every trio (mother, father, child) to identify *de novo* variants, as shown in Fig 10:

The expression above checks the genotype pattern along with genotype quality and limits to rare variants by INFO.gnomad_af < 0.001.

Expressions on families (including multigenerational) and arbitrary groups are supported so that, for example, expressions can be applied to tumor-normal pairs using tumor and normal labels.

### 3.5 VCF programming libraries

VCF programming libraries are mainly useful when direct calls to vcflib and bio-vcf command line tools proves too limited. The Bio* libraries, e.g., biopython [26], bioperl [27], bioruby [28] and R’s CRAN [29], contain VCF parsers that may be useful. But a first point of call may be vcflib itself as it is also a C++ programming library and in addition to being an integral part of the vcflib tools mentioned here is used by, for example, the freebayes variant caller [4].

**Fig 11:**
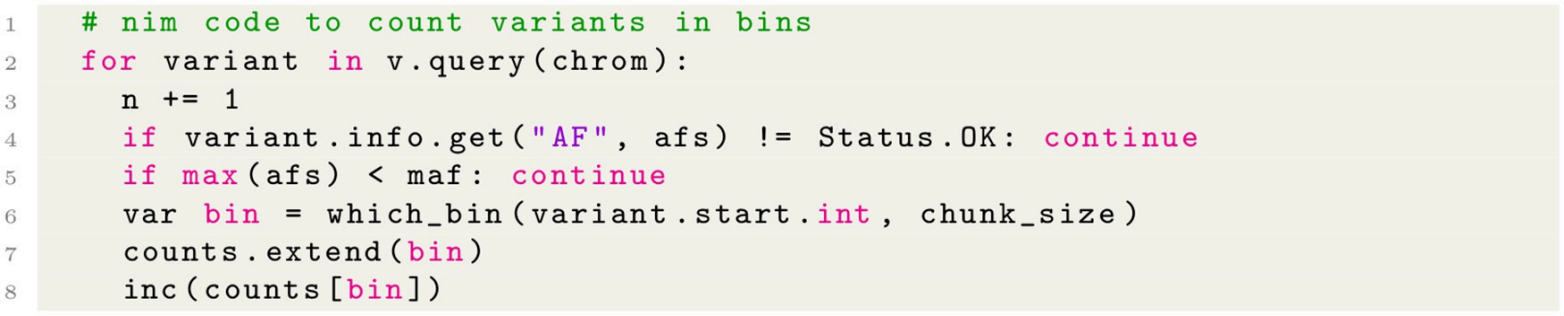


Of particular interest is the fast cyvcf2 library that was started in 2016 with htslib [14] bindings and is actively maintained by co-author Pedersen today [9]. Similar to bio-vcf it presents a DSL-type language that can be used in Python programming. Meanwhile hts-nim [10] contains bindings for the Nim programming language with similar syntax and functionality. For example a nim bin counter (part of hts-nim-tools/vcf-check [10]) can be written as shown in Fig 11:

Nim is a statically typed compiled language that looks very similar to Python and, because it transpiles to C, Nim has a much faster runtime and can link without overheads against C libraries such as vcflib and htslib.

Finally, another tool of interest, by the same author, is vcfanno; written in the Go language and allows annotations of a VCF with any number of INFO fields from any number of VCFs or BED files. vcfanno uses a simple conf file to allow the user to specify the source annotation files and fields and how they will be added to the info of the query VCF [30].

## 4 Discussion

Ten years is a long time in bioinformatics and the VCF file format is starting to show its age. Not only is the VCF format redundant and bloated with duplication of data, a more important concern is that the VCF format does not accommodate interesting complex genomic variations, such as complex and nested variants, such as superbubbles, ultrabubbles, and cacti [31–33]. An even more important shortcoming of VCF is that it always depends on a single reference genome, resulting in variant calling bias and missing out on variation not represented in the reference [33]. One solution is to work with multiple reference genomes, but comparing VCF files from different reference genomes is challenging—even for different versions of one reference genome.

To address such challenges the authors are actively working on pangenome approaches that store variation in a pangenome graph format, e.g. [33–39]. Pangenomes can incorporate multiple individuals and multiple reference genomes. Pangenomes can cater for very complex structural variation. Pangenomes are also efficient in storing information, including metadata, without redundancy. In effect, pangenomes cater for a ‘lossless’ view of all data at the population level. This largely differs from VCF-type data because, despite mentioned data size, a generated VCF implies a data reduction step—or data loss—that effectively disconnects variants from each other and related features, such as quality metrics. This means that rebuilding the original data from VCF files is virtually impossible. In contrast, with the newer pangenome formats it is possible to rebuild sequences independent of the underlying complexity of features. Having a full view of the data makes downstream analysis, such as population genotyping, more powerful with improved results, e.g. [33].

The VCF file format has become a crucial part of almost all sequencing workflows today. The design and presentation of the VCF file format can set the norm for designing future file formats [1, 2], but we can also learn from its mistakes. In this paper we wrote VCF ‘standard’ consistently between quotes because, even though there exists a standardization effort—now at VCFv4.3 [2]—VCF is flexible by design, alternative VCF standards are introduced (e.g. [15, 16]) and most tools take liberties when it comes to producing VCF files. Therefore all VCF parsers have to take a flexible approach towards digesting input data and ignore input data that is not understood.

We recognise that the success of a file format requires a crucial focus on having an early ‘standard’ that is both easy to understand and flexible enough to grow, in line with the success of other bioinformatics file formats, such as SAM/BAM [2] and GFF [40]. Biology is a fast moving field and it is impossible to predict how a file format is going to be used in a (near) future. The downside of such flexibility is that older software may not support features that were added later. One of the weakest aspects of the VCF format is its metadata: next to *ad hoc* metadata in records (see Fig 2), the header record requires specialized parsing and ignores existing ontologies.

Also for the VCF records, robust validation, error checking and correctness checking is virtually impossible. Great attention should therefore be paid to any amendments to an earlier standard to keep backward compatibility when possible. VCF and many other formats in bioinformatics use layered character separators as a grammar for defining a tree structure of data (see Fig 2). This type of format requires specialized *ad hoc* parsers for every format. In the future, when designing new formats, we strongly suggest to base a new format on existing standards such as JSON, JSON-LD and RDF web formats for storing hierarchical data and graph data respectively. Each of these formats has efficient storage implementations. A future format should also benefit from reusing existing ontologies or create and champion a new ontology, if one does not exist, so data becomes easily shareable, comparable and queryable and living upto FAIR requirements [41]. Not only are JSON, JSON-LD and RDF natively and efficiently supported by most computer languages, they are also more easily embedded in existing infrastructure, such as NoSQL databases.

### Software development and distribution practices

In this paper we present three types of tools that mirror three common approaches in bioinformatics towards large data parsing. First are vcflib Unix style command line tools where each tool does a small job [8]. Second are bio-vcf and slivar-style extremely flexible command line DSLs. And third are programming against libraries that can be called from programming languages, such as cyvcf2 and hts-nim, as well as vcflib and bio-vcf.

A wide range of solutions exist for VCF processing that make use of these three approaches and functional overlap is found between vcflib, bio-vcf, cyvcf2, the original vcftools [1], bcftools [6] and the existing Bio* programming libraries, such as biopython [26], bioruby [28] and biojava [42]. vcftools and bcftools provide annotation, merging, normalization and filtering capabilities that complement functionality and can be combined in workflows with vcflib and bio-vcf. These solutions together provide a comprehensive and scalable way of dealing with VCF data and every single tool represents a significant investment in research and software development. Therefore, before writing a new parser from scratch, we strongly suggest to first study the existing solutions. In the rare case a new tool is required it may be an idea to merge that with existing projects so everyone can benefit.

Once software is written, it is important software development and maintenance continues. In the biomedical sciences it is a clear risk for projects to get abandoned once the original author moves on to another job or other interests; partly due to a lack of scientific recognition, attribution and reward [43]. We note that with the pyvcf project, for example, this has happened twice and the github contribution tracker shows no more contributions by a project owner. This means no one is merging changes back into the main code repository and the code is essentially unmaintained. vcflib, bio-vcf and cyvcf2, in contrast, show a continuous adoption of code contributions thanks to the original authors encouraging others to take ownership and even release versions of the software. We also recognise the importance of creating small tools that can interact with each other following the Unix philosophy.

For overall adoption of software solutions it is important the tools and documentation get packaged by software distributions, such as Bioconda [44], Debian [45] and GNU Guix [46, 47]. Bioconda downloads are a good estimation of relative popularity because they tend to represent actual installations. vcflib, for example, was installed over 70,000 times through Bioconda by December 2021 (30,000 in 2021 alone). Note that vcflib is also an integral part of the freebayes variant caller with an additional 160,000 downloads through Bioconda (50,000 in 2021). Meanwhile bio-vcf was installed over 17,500 times through Bioconda by December 2021.

### Future work

Software development never stands still. With new requirements tools get updated. With the evolving VCF ‘standard’ and associated tooling for pangenomes and reference based approaches we keep updating our tools and libraries. We intend to add more documentation and regression tests. One recent innovation in vcflib is the generation of Unix ‘man’ pages from markdown pages and the --help output from every individual tool and script that also doubles as regression tests. This documentation is always up to date because when it goes out of sync the embedded tests fail. We think it will also be useful to add tool descriptions in the common workflow language (CWL) format [47–49]. CWL definitions allow easy sharing of tool components between sequencing workflows. The semantics of inputs and outputs are formally represented in a CWL workflow (a tool is one definition, the connection of tools another). The scenario will be to write a CWL tool definition that can be converted to documentation and running tests. One of the interesting features of CWL tool definitions is ‘type checking’ of tool calls, e.g. an error is shown if wrong parameters are used. Likewise CWL runners check return codes are more powerful than shell scripts.

Despite our criticism of VCF, VCF as a file format is likely to remain in use. To replace VCF most existing tools and workflows used in sequencing would have to be rewritten. Pangenome tools, in principle, are capable of producing reference guided VCF files from GFA graph structures. These tools guarantee compatibility with upstream and downstream analysis workflows. We predict that pangenome approaches will play an increasingly important role in sequence analysis and, at the same time, VCF processing tools will remain a crucial part of sequencing workflows for the forseeable future.
